# Increased threat learning after social isolation in human adolescents

**DOI:** 10.1098/rsos.240101

**Published:** 2024-11-13

**Authors:** E. Towner, K. Thomas, L. Tomova, S-J. Blakemore

**Affiliations:** ^1^ Department of Psychology, University of Cambridge, Cambridge, UK; ^2^ Cardiff University Brain Research Imaging Centre (CUBRIC), Cardiff University, Cardiff, UK

**Keywords:** social isolation, threat learning, loneliness, anxiety, adolescence

## Abstract

In animal models, social isolation impacts threat responding and threat learning, especially during development. This study examined the effects of acute social isolation on threat learning in human adolescents using an experimental, within-participant design. Participants aged 16–19 years underwent a session of complete isolation and a separate session of isolation with virtual social interactions, counterbalanced between participants, as well as a baseline session. At baseline and following each isolation session, participants reported their psychological state and completed a threat learning task in which self-report ratings and physiological responses to learned threat and safety cues were measured. Threat learning increased after both isolation sessions in two ways. First, participants found the learned threat cue more anxiety-inducing and unpleasant after isolation compared with baseline. Second, during threat extinction, electrodermal activity was partially elevated after isolation compared with baseline. Further, the results suggested that isolation influenced threat learning through state loneliness. Threat learning is central to threat-related disorders including anxiety, phobias, obsessive-compulsive disorder (OCD) and post-traumatic stress disorder (PTSD), and our findings suggest that isolation and loneliness in adolescence might increase vulnerability to the emergence of these disorders through increased threat learning.

## 1. Introduction

Social isolation and loneliness are growing issues worldwide [1,2]. Amid the COVID-19 pandemic, isolation (the objective state of being alone) and feelings of loneliness (the subjectively perceived lack of social connection; [3]) increased across geographic locations and diverse demographic groups [4]. Even before the pandemic, loneliness had been worsening in young adults (aged 18–29 years) since the late 1970s, according to a meta-analysis of 345 studies [5], with similar trends observed in adolescents aged 13–18 years [6,7]. Surveys indicate that loneliness levels are especially high among 16–24-year-olds, surpassing all other age groups in many countries [1]. Adolescents, defined as individuals aged 10–24 years [8], might be particularly prone to isolation-induced loneliness, as adolescence constitutes a period of social reorientation in which social connections become increasingly important [9,10].

Paradoxically, while being the loneliest age group, adolescents also report more social media use than other age groups [11]. Indeed, it has been proposed that the increase in loneliness among young people in recent years might even be due to the rise in digital media use and the concomitant decline in in-person social interactions [6,7]. However, the empirical evidence remains inconclusive about whether social media use alleviates or exacerbates loneliness [12,13], as virtual social interactions can enhance existing personal relationships and foster the development of new, positive and supportive connections [14].

Loneliness is associated with a host of negative physical and psychological outcomes including depression, anxiety, heart disease, substance abuse and premature mortality [15–17]. Of particular interest is the link between loneliness and anxiety. Anxiety can be conceptualized as a future-oriented state triggered by prospective threat [18]. Associative learning mechanisms, in which individuals learn to associate threats with otherwise neutral cues, are proposed to be crucial mechanisms in the aetiology of anxiety disorders [19]. The Evolutionary Theory of Loneliness [3] provides an explanation as to why loneliness might be linked with heightened responses to threat [20]. This theory suggests that social isolation induces an adverse biological signal (loneliness), which evolved to promote vigilance against danger (such as threats of predation or lack of resources) [3,21]. This loneliness-induced anxiety might thereby increase survival prospects by increasing threat vigilance.

In animal models, social isolation has been shown to increase threat responding (reactivity to potentially threatening events) demonstrated by increased anxiety-like behaviours in rodents (particularly when isolation occurs during development; [22,23]). In addition to threat responding, animal research also suggests that isolation can impact threat learning. For example, social isolation results in increased conditioned threat responses in rodents [23,24]. Further, socially isolated adolescent [25] and adult [26] rodents exhibit impaired threat extinction learning compared with socially housed conspecifics.

In humans, both chronic and state loneliness have been associated with altered threat processing, with studies finding links between loneliness and threat hypervigilance, particularly towards social threats, at the neural, behavioural and self-report levels [27–29]. Research investigating human Pavlovian threat learning has shown that young adults with high levels of chronic loneliness had a higher tendency for fear relapse (retention), or the return of a threat response after extinction (as measured by electrodermal activity) compared with individuals with low levels of chronic loneliness [30]. Another study found that men (average age 26 years) reporting high levels of chronic loneliness displayed hyperreactivity in the amygdala (a brain region associated with emotional responses including fear and anxiety; [31]) during fear conditioning compared with men reporting low levels of loneliness [32]. Further, high-chronic lonely men showed reduced amygdala habituation during repeated exposure to fearful faces [32], though this pattern was not found in women. Unlike the animal literature, in humans, there is a lack of experimental studies that directly manipulate social isolation and enable inferences that go beyond correlations between self-reported loneliness and threat learning. Experimental studies in humans can also elucidate whether there are different effects of social isolation on threat learning when participants are physically isolated but have access to virtual social interactions.

### 1.1. The current study

Here, we employed an experimental approach to investigate the effect of acute isolation, with and without virtual social interactions, on threat learning in human adolescents aged 16–19 years. We aimed to test the effects of isolation in a population for whom the consequences of loneliness are particularly important [9,33]. Participants underwent three sessions, starting with baseline and followed by two isolation sessions of up to 4 h. One isolation session (iso-total) involved isolation from all social interactions, while the other (iso-with-media) allowed for virtual social interactions only. At each session (baseline, iso-total and iso-with-media), threat learning was measured using self-report ratings (arousal and valence) and physiological measurements (electrodermal activity) during a Pavlovian threat learning paradigm. In this article, we focus on two research questions: (Q1) How does acute isolation impact threat learning (including acquisition, extinction and retention)? and (Q2) Do virtual social interactions alter the effects of acute isolation on threat learning? We hypothesized that isolation would heighten threat learning by increasing threat acquisition, decreasing threat extinction and increasing threat retention and that virtual social interactions might lessen the effects of isolation on threat learning.

## 2. Methods

This study is part of a larger project examining the effects of social isolation on cognition in adolescence (Open Science Framework (OSF)—https://doi.org/10.17605/OSF.IO/W5UM9; pre-registration—https://doi.org/10.17605/OSF.IO/KBGSV). Therefore, participants and study design are identical to those reported in a separate manuscript investigating the effects of acute isolation on reward responsiveness in human adolescents [34]. Data and electronic supplementary materials related to this manuscript can be found in a subcomponent of the larger study (OSF; https://doi.org/10.17605/OSF.IO/N9XP4). All study procedures, methodologies, hypotheses and analysis plans were pre-registered (deviations from the pre-registration have been outlined in the electronic supplementary material, appendix B).

### 2.1. Participants

As previously reported [34], this study included 40 participants (*M*_age_ = 17.1, s.d._age_ = 0.9, range_age_ = 16–19 years, 22 female). In order to increase our power to detect effects related to our experimental manipulation (social isolation), we aimed to minimize potential variation due to age-related differences. Therefore, we restricted the age range of participants to 16–19 years. Participants were recruited through advertisements (flyers and online) and local schools in Cambridge. Potential participants were first assessed for eligibility via a screening questionnaire (administered through Qualtrics and REDCap; see the electronic supplementary material, appendix G for details on software). To be deemed eligible, candidates had to be between 16 and 19 years of age, with no history of brain damage, no currently diagnosed mental health disorders and no metal implants in their body (as this study was part of a larger project involving an MRI scan). The study was conducted amidst the COVID-19 pandemic, hence a COVID-19 risk assessment formed a part of the exclusion criteria, disqualifying candidates with chronic illnesses (such as asthma), current health issues (or a positive COVID-19 test) and those who smoked. We wanted to avoid conflating effects of chronic and acute isolation and loneliness for experimental reasons and also to avoid exposing vulnerable individuals to isolation. Therefore, candidates living alone, those who recorded high chronic loneliness levels on the UCLA loneliness scale ([35]; a score greater than 50 was regarded as high, 1 s.d. above an adolescent sample mean), and/or those with significantly smaller social network sizes as compared with a previous adolescent sample ([36]; *n* = 40), were excluded. Social network size was estimated by the number of close friends and number of social interactions over the previous month, considering both in-person and virtual social interactions of a non-professional nature. Given the social distancing norms during the pandemic, the study set lower exclusion thresholds for social network size than used previously [37]. Candidates reporting fewer than two close friends and/or 10 social interactions within the last month (approx. 7 s.d. below the adolescent mean; [36]) were excluded. The Psychology Research Ethics Committee at the University of Cambridge reviewed the experimental procedures. Prior to the study, participants (and parents of participants under 18) provided informed consent. For their involvement in three sessions, participants were compensated between £107 and £127, depending on task performance.

### 2.2. Study design

**Figure 1.**
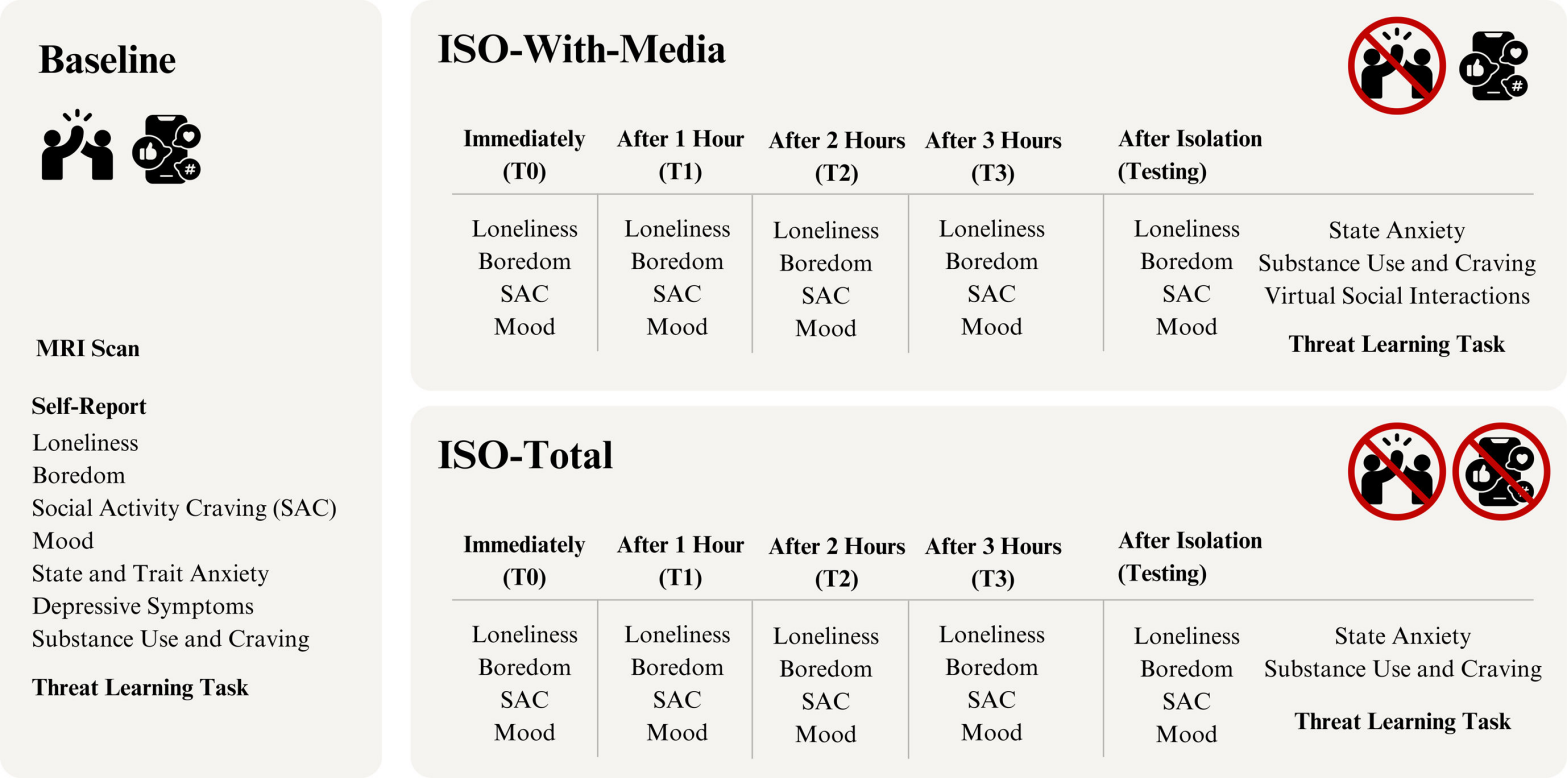
Study design. This figure illustrates the study design, including the components of the procedures relevant to this manuscript that were delivered at each session. We used a within-participant design, and the isolation sessions were counterbalanced between participants. The self-report questionnaires collected at each hour of isolation and at the beginning of testing are listed. For more information on the measures used, see the ‘Questionnaire’ section below. The threat learning task was administered at each session. Further details on the other behavioural tasks and the MRI scan administered in this study are described elsewhere (pre-registration: https://doi.org/10.17605/OSF.IO/N9XP4; [34]).

Participants took part in three experimental sessions (baseline, iso-with-media and iso-total; figure 1).

Each session was separated by a minimum of 24 h (days between sessions ranged from 2 to 125 (*M* = 32.5, s.d. = 27.51) across participants). The first session was a baseline, consisting of MRI scans and testing (which included tasks, questionnaires and physiological measurements). After baseline, participants returned for two sessions of isolation (order counterbalanced between participants) followed by testing. One session involved up to 4 h of complete social isolation (iso-total), where participants had no access to any form of social interaction. The other session involved up to four hours of social isolation, with access only to virtual social interactions (iso-with-media). Following methods from Tomova *et al.* [37], we aimed to induce a temporary subjective sense of state loneliness among adolescent participants. Two hours of isolation was sufficient to increase self-reported state loneliness in this population according to piloting and reanalysis of a subset of data from Tomova *et al.* [37]. In the current study, each isolation session lasted a minimum of 3 h and 30 min. An additional 0–30 min of isolation, in 5 min increments, was randomly assigned to each session. This was done to prevent participants from being able to predict the end of their isolation in either session. Therefore, all sessions lasted between 3 h and 30 min and 4 h. The average isolation duration was similar across sessions (iso-with-media: *M* = 3 h 47 min, s.d. = 10 min; iso-total: *M* = 3 h 46 min, s.d. = 11.20 min; *t*(39) = 0.30, *p* = 0.765).

Participants could spend the isolation period as desired (excluding sleeping). The isolation room contained an armchair, desk, office chair, desktop computer, physiological hardware, fridge with food and beverages and non-social materials (puzzles, sudoku books, digital and analogue games). Participants could also bring their own non-social items (crafts, textbooks and writing materials). For the iso-with-media session, materials with social components (music and novels) were allowed and participants retained their electronic devices and had Internet access. In the iso-total session, participants were not allowed to bring their own electronic devices but retained access to a messaging application (Slack; in case of emergency) and our experimental software via a desktop computer. Participants received a detailed PDF of instructions, and they completed questionnaires hourly. During isolation and subsequent testing, a live camera feed (without audio) allowed a researcher to periodically monitor participants. All participants underwent all three experimental conditions. The sequence of conditions was pseudo-randomly assigned per participant, with the baseline always being the first and with each sequence having approximately the same likelihood in the full sample. This design allowed for an analysis of the effects of both forms of isolation, using a baseline that had not been influenced by any previous isolation experience. It also enabled a direct comparison between the effects of complete isolation and isolation with access to virtual interactions.

#### 2.2.1. Questionnaires

At the start of testing in each of the three sessions, we gathered self-reported data on state loneliness, social activity craving (i.e. how much participants wanted to engage in a social activity), boredom and mood (evaluated with the Positive and Negative Affect Schedule—PANAS; [38]). At baseline only, we also collected data on trait anxiety (using the trait subscale of the State Trait Anxiety Index (STAI); [39] and depression (utilizing the Center for Epidemiological Studies Depression (CES-D) scale; [40]). In both the iso-total and iso-with-media sessions, we also evaluated state loneliness, social activity craving, boredom and mood at hourly intervals during the session. State anxiety was collected once in all three sessions (at the start of testing) using the state subscale of the STAI [39].

Participants completed a questionnaire about their virtual social interactions during the isolation period after the iso-with-media session. They estimated the percentage of time they spent engaging in virtual social interactions during the session (ranging from 0% to 100%). Participants were also asked to specify the primary virtual social interaction method(s) (e.g. texting/messaging, voice calls, video calls, commenting/posting, gaming and others) and platform(s) (e.g. Instagram, Facebook, Facebook Messenger, Snapchat, TikTok, Twitter, WhatsApp and others) they used during the session. Additionally, participants identified their virtual interaction partners (e.g. friends, family, acquaintances, romantic partners and others). Participants could select multiple options and, if they selected ‘other’, were asked to elaborate.

#### 2.2.2. Threat learning task

**Figure 2.**
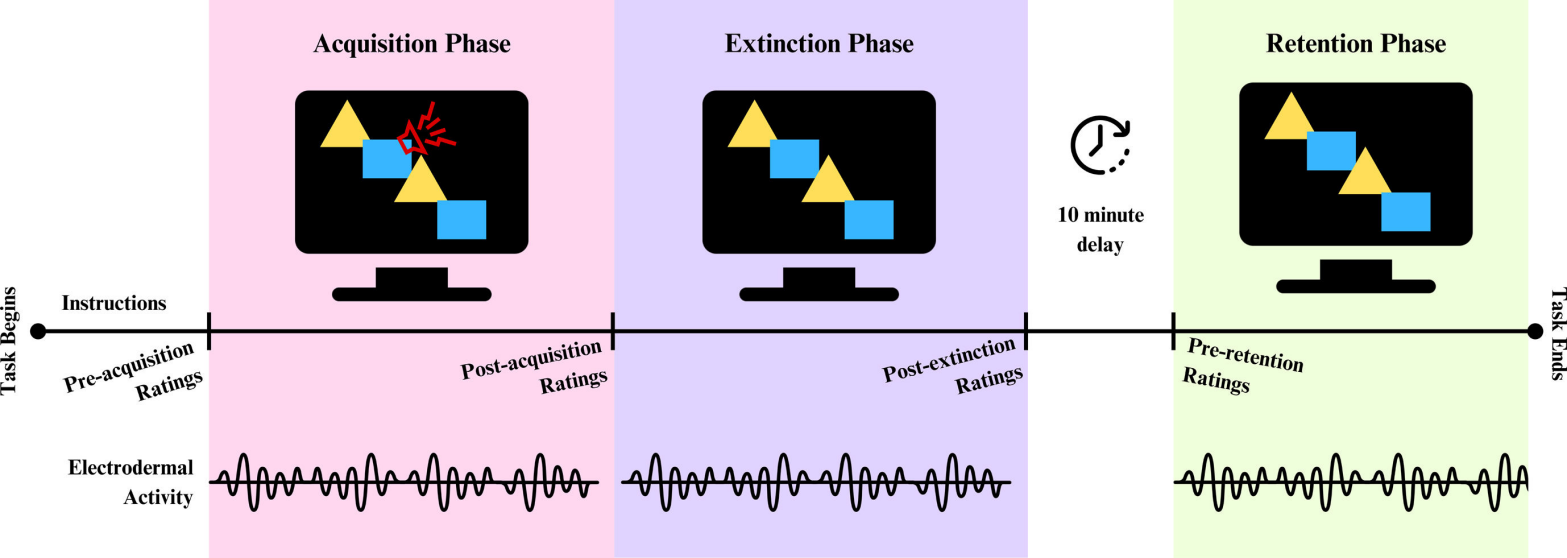
Threat learning task design. This figure illustrates the critical phases of the threat learning task. Participants provided self-report ratings (valence and arousal) at each tick mark on the timeline. Electrodermal activity was recorded continuously throughout the three phases. During acquisition, participants learned that one of the two neutral shapes (threat cue) was sometimes paired with an aversive sound. During extinction, participants learned that the threat cue was no longer paired with the aversive sound. After a 10 min delay (that included an unrelated task), participants again saw the cues with no aversive sound (retention).

We measured threat learning using a Pavlovian threat learning paradigm (programmed using PsychoPy) in which participants learned to associate neutral shapes with an aversive sound (a metal screech). Threat responses were measured via self-report ratings and physiologically via electrodermal activity across three phases (acquisition, extinction and retention; figure 2). Pre-acquisition responses were also collected for self-report ratings. One shape (such as a blue rectangle) served as a threat cue (CS+) and another (such as a yellow triangle) as a safety cue (CS−). There were six shape stimuli in total which were fully counterbalanced between participants and sessions, with participants seeing a different pair of shape stimuli in each session (to avoid carry-over effects).

In the baseline session, each participant first underwent a sound calibration: the aversive sound was initially played to each participant through headphones. Participants were given the opportunity to change the volume to make the sound ‘unpleasant, but not painful’, and this volume was recorded and used in all subsequent sessions. The researcher then explained to the participant how to attach electrodes to their left middle and index fingers to measure electrodermal activity and start the recording and task presentation software. The researcher monitored the application and execution of this procedure. This meant that participants could set up electrodermal activity recordings and self-administer the task independently at the following isolation sessions. Written instructions were also provided at each session. We used a Biopac (MP36R) recording system together with AcqKnowledge software to amplify and record electrodermal activity (electrodermal activity channel sample rate (2000 samples per s), acquisition sampling rate (2000 samples per s) and gain (×2000); [41]). We used a Biopac electrodermal activity finger transducer (SS3LA) with isotonic gel (GEL101).

#### Pre-acquisition

Once the task began, participants were given instructions via a computer monitor. First, they were shown the two shapes (threat and safety cues) and asked to judge the valence and arousal of each shape (pre-acquisition ratings). For valence, participants were asked ‘How unpleasant/pleasant do you find this shape?’ (from (1) very unpleasant to (9) very pleasant). For arousal, participants were asked ‘How anxious does this shape make you feel?’ (from (1) not anxious to (9) very anxious). Rating scale tick marks were accompanied by figures from the Self-Assessment Manikin [42], and a pictographic scale was used to assess affective responses on dimensions of valence and arousal. There was no time restriction for participant ratings.

#### Acquisition

After pre-acquisition ratings, participants completed an acquisition (i.e. learning) phase. Participants were told they would see different coloured shapes and that some of the shapes might be followed by a sound. There were 24 trials in which a shape (either the threat or safety cue) was presented on the screen for 2 s (with a 7 s inter-trial interval). On 50% of threat cue trials, the shape co-terminated with the aversive sound for the last 500 ms of that trial (16 threat cue trials total; eight paired with aversive sound and eight without). The safety cue (eight trials) was never accompanied by the aversive sound. Participants were not informed which shape would be followed by the sound. They were told that, after some time, a grey dot would appear in the centre of the shape, and to press the spacebar with their right hand as quickly as possible upon seeing the grey dot. This dot appeared after 1.5 s on each trial and served as an attention check. Immediately after the acquisition phase, participants again rated the valence and arousal of both shapes. As a second attention check at the end of the acquisition phase, participants were then asked to indicate which shape was followed by the sound. Data were not excluded from the analysis based on either attention check, but post hoc sensitivity analyses concluded that excluding data based on these attention checks did not change the overall pattern of results (see the electronic supplementary material, appendix F).

#### Extinction

Extinction measures the ability to suppress previously learned threat associations once they are no longer predictive of threat. Therefore, during the extinction phase, the aversive sound was no longer paired with either stimuli. Participants were shown identical instructions as in the acquisition phase. The extinction phase consisted of 16 trials (eight threat cues and eight safety cues). After extinction, participants again rated the valence and arousal of both shapes.

#### Retention

After a 10 min delay, during which participants completed an unrelated social influence task [43,44], retention was assessed. In the retention phase, we measured the threat response after a period of time had elapsed in order to assess long-term threat responding. Participants were shown identical instructions as in the acquisition and extinction phases. Participants rated the valence and arousal of both shapes then underwent 16 trials (eight threat cues (no longer paired with the aversive sound) and eight safety cues). Finally, at the end of the retention phase, participants were asked to rate the aversive sound (US) in arousal and valence.

#### Design considerations

In order to avoid problems with concentration or fatigue, we kept the task as short as possible while still following design recommendations [45,46]. To reduce the probability that participants would deduce the structure of the task (repeated at baseline, iso-with-media and iso-total), we included a number of trials before the extinction phase began and after the retention phase had ended in which the threat cue was paired with the aversive sound. However, these trials were not included in the analyses—and their placements (at the beginning of the extinction phase and at the end of the retention phase) were chosen to avoid contamination with other phases. For full information regarding the task design, see the electronic supplementary material, appendix C.

#### 2.2.3. Other tasks

Participants completed several other tasks as a part of this study, including a reward-seeking task (effort-based decision-making), reward learning task (reinforcement learning), a cognitive control task (go/no-go) and a social influence task (see pre-registration: https://doi.org/10.17605/OSF.IO/N9XP4). Findings from other tasks are reported elsewhere [34]. The acquisition and extinction phases of the threat learning task were completed after the reinforcement learning task, the effort-based decision-making task and the go/no-go task. Participants completed the social influence task between the extinction and retention phases of the threat learning task, as described above.

### 2.3. Data analysis

#### 2.3.1. Data preprocessing—electrodermal activity

Data were preprocessed using standard parameters in NeuroKit2 [47]. This involved removing noise and smoothing the signal (using a low-pass filter with a 5 Hz cut-off frequency and a fourth order Butterworth filter) before decomposing the signal into phasic and tonic components (by passing the cleaned data through a high-pass filter with a cut-off frequency of 0.05 Hz). After preprocessing, both the processed and raw electrodermal activity data were visually inspected by one rater for quality control. The quality of the data was good, and no participants or trials were removed from the data. Epochs were identified using event markers for each condition. Peaks were identified in the phasic component of the processed electrodermal activity signal using the default neurokit settings. Responding during threat learning is, by definition, temporally dynamic [48,49]. Therefore, electrodermal activity (peak amplitude) was averaged per condition (i.e. threat, safety) for the last half of the acquisition and extinction phase (once learning occurred), and first half of the retention phase (before additional extinction occurred) in order to capture the relevant response of interest. These time bins were pre-registered and selected based on prior literature [45]. In order to separate the learned response from the response to the aversive sound itself, threat trials paired with the aversive sound were excluded from analyses of electrodermal activity.

#### 2.3.2. Confirmatory analyses

#### Threat learning

To compare differences in threat responses associated with our experimental manipulation, we used linear mixed effects models. We used linear mixed effects models due to the within-participant structure of our data (data were nested within participants). Effects of interest were estimated as the fixed effect of the interaction between session (baseline, iso-with-media and iso-total), phase (pre-acquisition, acquisition, extinction and retention) and cue (threat and safety) on each response (arousal, valence and electrodermal activity; note, there was no pre-acquisition phase for electrodermal activity). ‘Participant’ was included as a random effect, allowing intercepts to vary between participants (accounting for individual variation in participants’ average responses). We did not include random slopes in our models due to our mixed factorial design (in designs with a single observation per unit per cell, random slope variance cannot be distinguished from random error variance and thus random slopes should not be included) [50]. The formula for these models was ‘response = session × phase × cue + (1 | participant)’. ANOVA tests of fixed effects of the categorical predictors and interactions were performed on each model. Interactions were interpreted using post hoc pairwise comparisons of the contrasts between threat responding (responses to the threat and safety cue at each session and phase) and threat discrimination (the difference in response between the threat and safety cue at each session and phase) using estimated marginal means and errors. Post hoc pairwise comparisons were corrected using the Bonferroni method for multiple comparisons (three sessions—baseline, iso-total and iso-with-media; three responses—arousal ratings, valence ratings and electrodermal activity) for an adjusted alpha level of 0.005 (0.05/9). These models allowed us to assess threat learning across phases of the task as well as to compare sessions (to determine whether virtual social interactions remediated the effects of isolation on threat learning). Confirmatory analyses were carried out using R.

#### 2.3.3. Exploratory analyses

#### Emotions and mood

In this article, we sought to explore the degree to which participants’ emotions and mood during isolation (state loneliness, boredom, social activity craving, positive and negative moods and state anxiety) act as ‘mechanisms’ (as defined in [51]) by which isolation affects threat learning. To help with clarity and interpretation of the exploratory analyses, here we report simplified analyses of the effects of isolation on these state variables (but note that these results have been previously reported as confirmatory analyses in [34]). For these simplified analyses, we used linear mixed effects models to estimate the fixed effect of session on each state variable. ‘Participant’ was included as a random effect, allowing intercepts to vary between participants. For state variables that were collected hourly during the isolation sessions (state loneliness, boredom, social activity craving, positive and negative moods), ratings at hour 3 were used as the ultimate measure of experimentally induced emotions and mood, as this captured participants’ affective state after a substantial period of isolation but before we told them the isolation period was over. For state variables collected once (state anxiety), ratings at the final hour (just before the experimental tasks) were used. The formula for these models was ‘state variable = session + (1 | participant)’. ANOVA tests of fixed effects of session were performed on each model. Session effects were interpreted using post hoc pairwise comparisons of the contrasts between sessions using estimated marginal means and errors. Post hoc pairwise comparisons were corrected using the Bonferroni method for multiple comparisons (three sessions—baseline, iso-total and iso-with-media) for an adjusted alpha level of 0.016 (0.05/3).

#### Mechanisms

Causal processes are typically complex, involving various underlying mechanisms. Several processes might simultaneously mediate or ‘carry’ the effect of isolation on threat learning. Parallel mediation models respect such complexities and allow for the comparison of the size of indirect effects through different mediators. To quantify the degree to which the six state variables act as ‘mechanisms’ by which isolation affects threat learning, we employed a path-analytic framework to perform a two-condition within-participant statistical parallel mediation analysis between the iso-total and iso-with-media sessions [51]. Two conditions (iso-total and iso-with-media) were included in these analyses (baseline was not). This was because the order of the two isolation sessions was counterbalanced between participants and identical in procedure apart from the experimental manipulation (whether participants were allowed access to virtual social interactions or not). In contrast, the baseline session was always conducted first and did not include an experimental manipulation.

We used the MEMORE macro [51] in SPSS to estimate parallel multiple mediation between the two isolation sessions and threat learning (separately for each phase and cue (including the threat cue, safety cue and difference between the two—threat discrimination)). We entered each state variable as a mediator simultaneously (state loneliness, boredom, social activity craving, positive mood, negative mood and state anxiety). Corresponding to the emotions and mood analyses above, for state variables that were collected hourly (state loneliness, boredom, social activity craving, positive and negative moods), ratings at hour 3 were used as the ultimate measure of experimentally induced emotions and mood. For state variables collected once (state anxiety), ratings at the final hour (just before the experimental tasks) were used. We focused on estimates of the indirect effects (i.e. how much isolation influenced threat learning through each state variable). We did not derive s.e. and *p*-values of the indirect effects as the sampling distribution of indirect effects is not typically normal [52,53]. Instead, we constructed 95% CIs using the percentile bootstrap method with 10,000 bootstrap samples [51]. We interpreted confidence intervals that did not overlap with zero as meaningful [51]. By using parallel mediation, we were able to estimate the indirect effect of each state variable while simultaneously controlling for other state variables (i.e. the effects are not confounded with the effects of other state variables).

#### 2.3.4. Sample size rationale

Prior to pre-registration, we conducted a power analysis for our experimental manipulation (the ability for acute social isolation to induce feelings of loneliness) from pilot data (a subset of data from [37] involving 18–24 year olds). In this pilot data, short-term isolation affected feelings of loneliness (using a self-report loneliness scale ranging from 0 to 100) with an effect size (Cohen’s *d*) of 0.47. Our power analysis showed that 38 participants were required to detect this medium effect size to achieve a power of 0.80 (1 − beta) at an alpha of 0.05.

Regarding the statistical power for our threat learning analyses, no previous study had examined the effects of experimental isolation on threat learning in human adolescents from which we could have based effect-size estimates. Importantly, however, we utilized a within-participant design (where each factor in the three-way interaction was also a within-participant factor). This type of within-participant design requires only one-third of the number of participants as a between-participants design to achieve similar statistical power [54].

## 3. Results

### 3.1. Confirmatory analyses

#### 3.1.1. Threat learning

**Figure 3.**
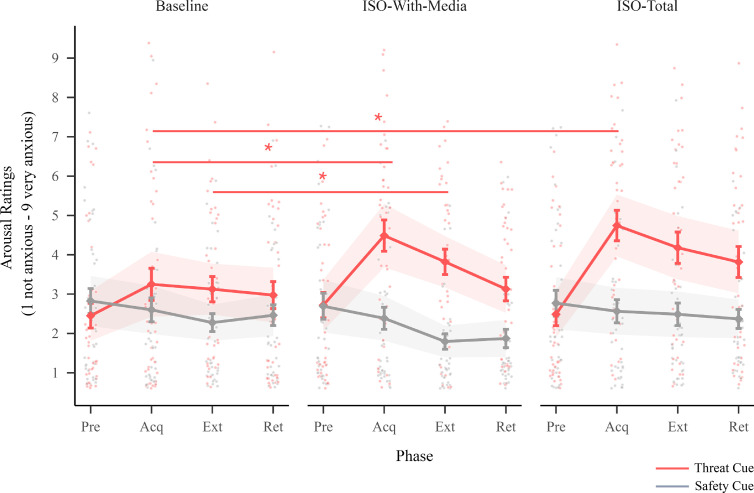
Arousal ratings. This figure illustrates arousal ratings for the threat and safety cue across sessions (baseline, iso-with-media and iso-total) and phases (Pre = pre-acquisition, Acq = acquisition, Ext = extinction and Ret = retention). Points indicate individual participant responses, diamonds indicate the estimated marginal means, bars indicate standard errors of the estimated marginal means and ribbons indicate the 95% CIs around the estimated marginal means. Lines (with stars) indicate a significant difference in post hoc pairwise comparisons (contrasting session effects for each cue at each phase; Bonferroni corrected for multiple comparisons (adjusted alpha = 0.005); see the electronic supplementary material, tables A8–A10 and A12–A14 for the full list of post hoc comparisons).

For arousal ratings, there was a two-way interaction between session and cue (F(2,877)=8.63,p<0.001) and between cue and phase (F(3,877)=17.19,p<0.001) (figure 3; electronic supplementary material, table A1). Post hoc comparisons (contrasts of threat responding) showed increased arousal ratings (higher anxiousness) to the threat cue after acquisition in both isolation sessions compared with baseline (electronic supplementary material, tables A8 and A9). Further, arousal ratings to the threat cue after extinction remained elevated after iso-total compared with baseline. Post hoc comparisons (contrasts of threat discrimination) showed a greater difference between the threat cue and safety cue after acquisition in both isolation sessions compared with baseline (electronic supplementary material, tables A12 and A13).

**Figure 4.**
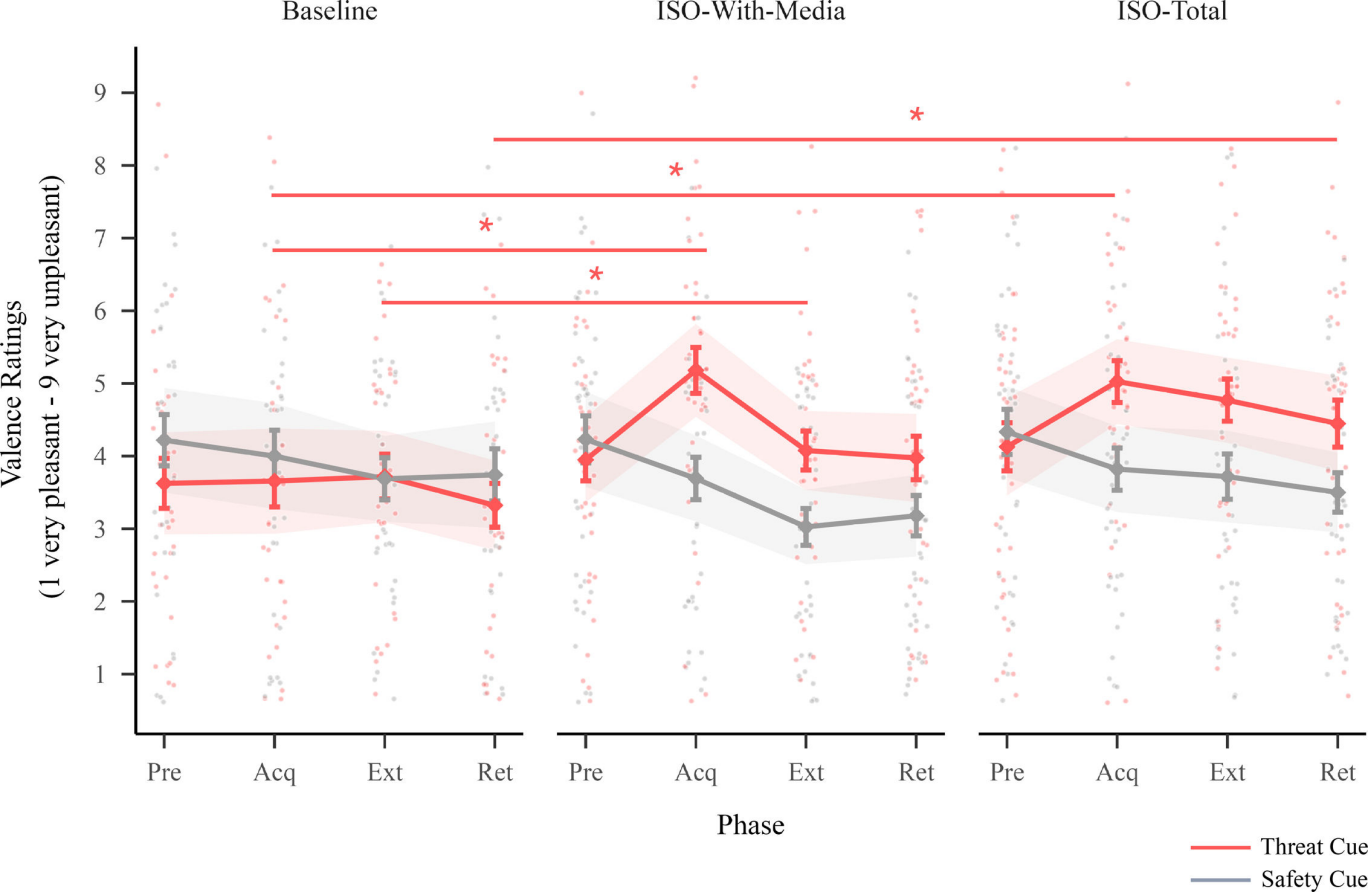
Valence ratings. This figure illustrates valence ratings for the threat and safety cue across sessions (baseline, iso-with-media and iso-total) and phases (Pre = pre-acquisition, Acq = acquisition, Ext = extinction and Ret = retention). Points indicate individual participant responses, diamonds indicate the estimated marginal means, bars indicate standard errors of the estimated marginal means, and ribbons indicate the 95% confidence intervals around the estimated marginal means. Lines (with stars) indicate a significant difference in post hoc pairwise comparisons (contrasting session effects for each cue at each phase; Bonferroni corrected for multiple comparisons (adjusted alpha = 0.005); see the electronic supplementary material, tables A8–A10 and A12–A14 for the full list of post hoc comparisons).

For valence ratings, there was a two-way interaction between session and cue ($F_{2,813}=12.68,p<0.001$) and between cue and phase ($F_{3,813}=7.11,p<0.001$) (figure 4; electronic supplementary material, table A3). Post hoc comparisons (contrasts of threat responding) showed increased valence ratings (higher unpleasantness) to the threat cue after acquisition in both isolation sessions compared with baseline (electronic supplementary material, tables A8 and A9). Valence ratings to the threat cue after extinction also remained elevated after iso-total compared with baseline. Further, valence ratings to the threat cue at retention remained elevated after iso-total compared with baseline. Post hoc comparisons (contrasts of threat discrimination) showed a greater difference between the threat cue and safety cue after acquisition in both isolation sessions compared with baseline (electronic supplementary material, tables A12 and A13). There was also a greater difference between the threat cue and safety cue at retention after iso-total compared with baseline.

**Figure 5.**
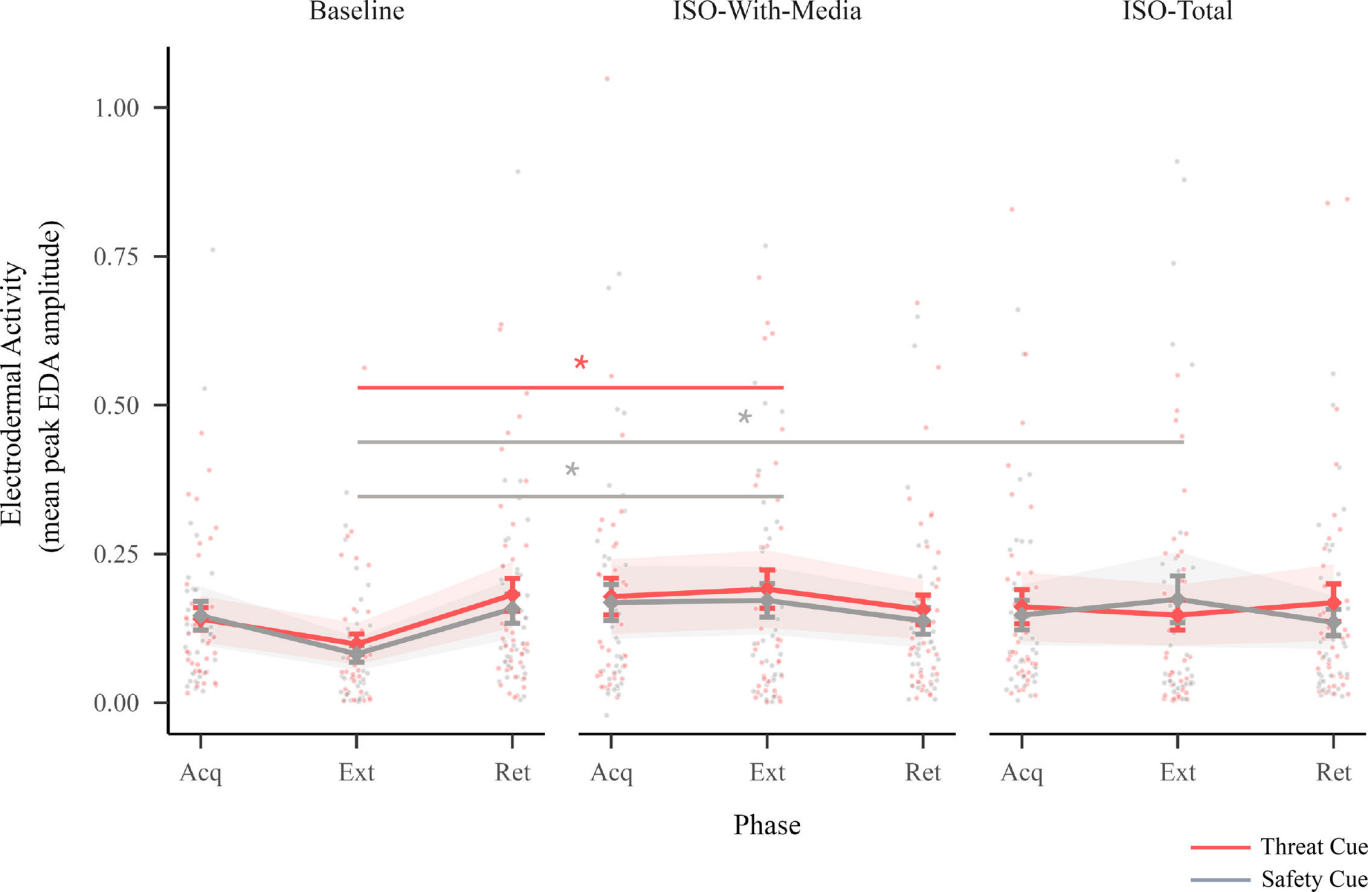
Electrodermal activity. This figure illustrates electrodermal activity for the threat and safety cue across sessions (baseline, iso-with-media and iso-total) and phases (Acq = acquisition, Ext = extinction and Ret = retention). Points indicate individual participant responses, diamonds indicate the estimated marginal means, bars indicate standard errors of the estimated marginal means, and ribbons indicate the 95% CIs around the estimated marginal means. Lines (with stars) indicate a significant difference in post hoc pairwise comparisons (contrasting session effects for each cue at each phase; Bonferroni corrected for multiple comparisons (adjusted alpha = 0.005); See the electronic supplementary material, tables A8–A10 and A12–A14 for the full list of post hoc comparisons).

For electrodermal activity, there was a two-way interaction between session and phase ($F_{4,645}=4.38,p=0.002$) (figure 5; electronic supplementary material, table A5). Post hoc comparisons (contrasts of threat responding) showed increased electrodermal activity to the threat cue during extinction in the iso-with-media session compared with baseline (electronic supplementary material, table A8). Further, electrodermal activity to the safety cue during extinction was also elevated in both isolation sessions compared with baseline (electronic supplementary material, tables A8 and A9).

Prior to beginning the threat learning task (pre-acquisition), there were no differences in arousal ratings or valence ratings to the threat or safety cue (or in the difference between the threat and safety cue) between any of the sessions (there was no pre-acquisition phase for electrodermal activity; electronic supplementary material, tables A8–A10 and A12–A14). The two isolation sessions (iso-with-media and iso-total) did not significantly differ from each other in threat responding or threat discrimination for any of the reported results above (electronic supplementary material, tables A10 and A14). There were no significant differences in ratings (arousal ratings nor valence ratings) or electrodermal activity to the aversive sound alone (unconditioned stimulus) between the three sessions (see the electronic supplementary material, appendix E). Further, sensitivity analyses provided no evidence that order effects contributed to any of the results (see the electronic supplementary material, appendix F).

#### 3.1.2. Emotions and mood

Detailed results regarding how state variables changed across the duration of isolation were reported previously [34]. We repeat a summary of the results here to help with clarity and interpretation of the exploratory mediation analysis below.

**Figure 6.**
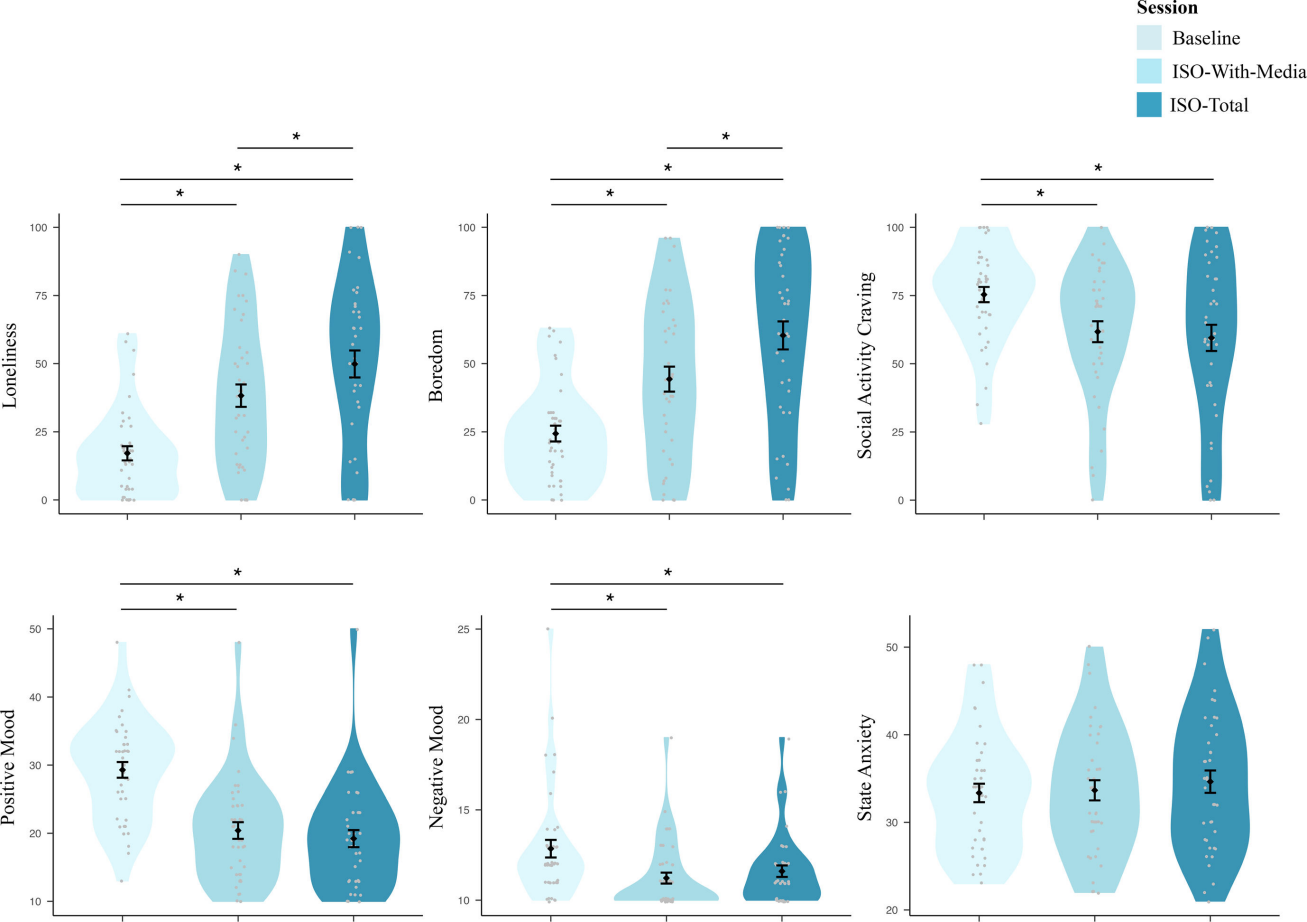
Emotions and mood. The effects of isolation on participants’ emotions and mood (state loneliness, boredom, social activity craving, positive and negative mood and state anxiety) were previously reported [34]; this figure shows a simplified version of the already-reported results). Points indicate individual participant responses, violins show the estimated data distributions, diamonds represent the estimated marginal means, and bars indicate standard errors around the estimated marginal means. Lines (with stars) indicate a significant difference in post hoc pairwise comparisons of estimated marginal means (Bonferroni corrected for multiple comparisons (adjusted alpha = 0.016); see the electronic supplementary material, tables A18–A20 for the full list of post hoc comparisons).

For loneliness ($F_{2,78}=26.03,p<0.001$), boredom ($F_{2,77}=28.24,p<0.001$), social activity craving ($F_{2,77}=13.34,p<0.001$), positive mood ($F_{2,76}=51.30,p<0.001$) and negative mood ($F_{2,77}=7.67,p=0.001$), there was an effect of session (figure 6; electronic supplementary material, table A15). There was no effect of session on state anxiety ($F_{2,77}=0.40,p=0.670$). Post hoc comparisons showed that after a period of isolation, participants reported higher levels of loneliness and boredom and lower levels of positive mood, negative mood and social activity craving at both isolation sessions compared with the baseline session (figure 6; electronic supplementary material, tables A18 and A19). Further, loneliness and boredom were elevated after the iso-total compared with the iso-with-media session. There were no differences between any of the sessions for state anxiety. State variable measurements were taken just prior to testing at the baseline session and after a period of isolation for the iso-with-media and iso-total sessions.

### 3.2. Exploratory analyses

#### 3.2.1. Mechanisms

**Figure 7.**
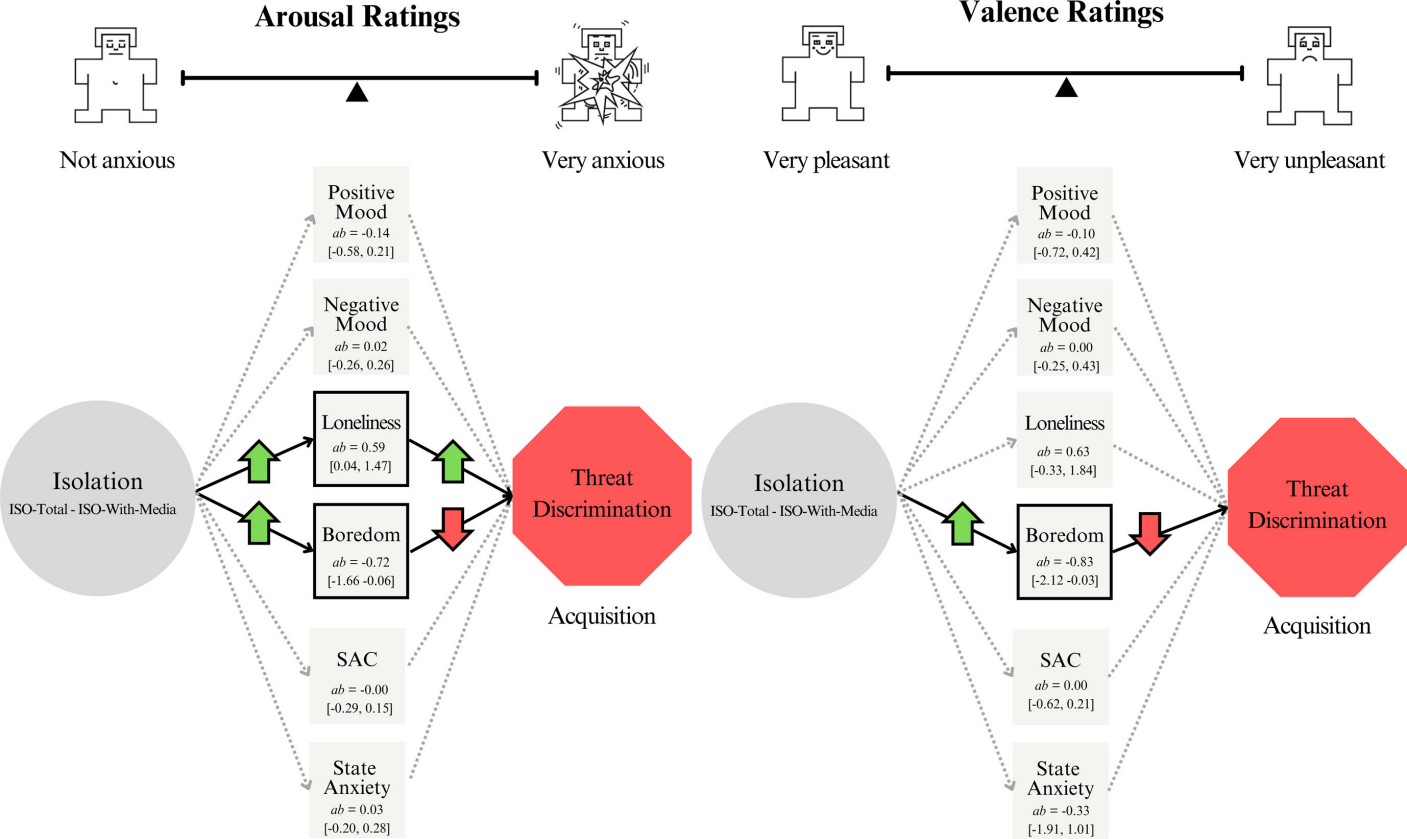
Mechanisms. This figure reports the results of the parallel multiple mediation (i.e. to what extent did increased isolation (iso-total – iso-with-media) influence threat learning (threat discrimination) through each state variable, while controlling for all other state variables) after acquisition. The left figure is for arousal ratings. The right figure is for valence ratings. Estimates (ab) are of the indirect effects. The 95% CIs around the indirect effects are reported in brackets. We interpreted confidence intervals that did not overlap with zero as meaningful (solid arrows and squares with black outlines; arrows indicate direction of the effect).

The path-analytic framework (see Methods for details) demonstrated that isolation influenced threat learning through state loneliness and boredom (figure 7) but not through other measured state variables (social activity craving, positive mood, negative mood or state anxiety). Notably, isolation influenced threat learning through state loneliness and boredom in opposite ways. Increased state loneliness in the iso-total condition (compared with the iso-with-media session) was associated with increased threat discrimination between the iso-total and iso-with-media sessions, as measured by arousal ratings after acquisition (while controlling for all other state variables; ab = 0.59, 95% bootstrap CI [0.04, 1.47]). In contrast, increased boredom in the iso-total compared with iso-with-media session was associated with decreased threat discrimination between the iso-total and iso-with-media sessions, as measured by arousal after acquisition (while controlling for all other state variables; ab = −0.72, 95% bootstrap CI [−1.66, −0.06]). Increased boredom in the iso-total (compared with iso-with-media session) was also associated with decreased threat discrimination between the iso-total and iso-with-media sessions, as measured by valence ratings after acquisition (while controlling for all other state variables; ab = −0.83, 95% bootstrap CI [−2.12, −0.03]). The full set of results are reported in the electronic supplementary material, appendix H.

## 4. Discussion

In this experimental study, we investigated the effects of social isolation, both with and without access to virtual social interactions, on threat learning in human adolescents (aged 16–19). Participants were tested at baseline and after they underwent two sessions of experimental isolation: one of total social isolation and another of social isolation with access to virtual social interactions (counterbalanced). We measured threat learning using a classical Pavlovian threat conditioning task, assessing both explicit (self-report ratings) and implicit (electrodermal activity) threat responses. We also measured self-reported psychological state (loneliness, boredom, social activity craving, mood and anxiety).

Our results showed that social isolation, irrespective of access to virtual interactions, led to heightened threat learning. Adolescents demonstrated marked increases in explicit threat discrimination (i.e. the difference between the threat and safety cue) after both isolation sessions compared with baseline, as evidenced by self-report responses. This increased threat discrimination was evident after the acquisition phase and remained partially elevated during the extinction and retention phases. Implicit responses, as measured by electrodermal activity, showed some evidence of elevation during extinction in both isolation sessions compared with baseline. Though we did not observe isolation effects on electrodermal activity during acquisition, prior studies investigating threat learning have shown that discrepancies often arise between explicit responses (self-reported ratings) and implicit responses (electrodermal activity) [55,56]. Further, past research suggests that threat learning involves two separate brain circuits [57,58]. The first an ‘implicit’ affective learning system [58] and the second an ‘explicit’ cognitive learning system, tied to the acquisition of knowledge about threats and the conscious experience of fear [59,60]. Future research investigating the neural mechanisms underlying threat learning after social isolation could provide valuable insights about the divergence of explicit and implicit responses.

Relatedly, in contrast to self-report responses, electrodermal activity was partially elevated to both the threat and safety cue. The inverse of threat learning (i.e. learning that a cue predicts an aversive stimulus) is safety learning. In our task, this translated to learning that one of the cues predicted the absence of the aversive stimulus. Threat and safety learning are related but distinct phenomena. Researchers have posited that learning about safety is more cognitively demanding than learning about threat [61], that safety learning might be differentially affected by exposure to stress, and that the effect of increased threat responses to safety cues is robust across anxiety disorders [62,63]. Age-related differences in threat generalization (i.e. the broadening of the threat response beyond the original threat stimulus) have also been found, with adolescents reporting less discrimination between threat and safety cues than adults [64]. In our study, whereas self-report responses indicated increased explicit threat discrimination after acquisition, the finding that electrodermal activity was elevated to both threat and safety cues might suggest generalization of implicit, physiological threat responses during extinction. Further research is needed to investigate these dissociations and their potential implications.

Overall, the current study replicates findings observed in animal (rodent) models, where increased threat conditioning and diminished threat extinction have been observed following social isolation [23–25]. Our results replicate these findings in human adolescents. Furthermore, our data bridge the gap between animal research and previous human studies, which have indicated that individuals with pre-existing chronic loneliness tend to exhibit higher rates of fear relapse in threat learning paradigms similar to the one used in this study [30]. Notably, in our study, even a short period of experimentally induced social isolation led participants to display enhanced threat learning compared with at baseline. This helps clarify potential ambiguities regarding the direction of causality in prior non-experimental human research (i.e. those that found correlations between chronic loneliness and threat learning), positioning social isolation as a plausible causal contributor to heightened threat vigilance.

Self-reported state loneliness increased across the duration of experimental isolation in this study and was higher after total isolation compared with isolation with access to virtual social interactions [34]. Our parallel mediation analysis demonstrated that state loneliness exerted a significant influence on explicit threat learning in our experiment. Participants who felt a heightened sense of loneliness during the study learned to discriminate between threat and safety cues more robustly. When accounting for boredom and other state variables as parallel mediators, we were able to isolate an indirect positive effect of isolation on explicit threat learning through state loneliness.

While we propose loneliness as a potential mechanism underlying the impact of isolation on threat learning, we acknowledge that we cannot make definitive causal claims about any mediators. Further, the effects of acute and chronic loneliness on threat learning might be different [65]. In other domains, it has been posited that acute and chronic loneliness might have opposite effects. For example, acute loneliness might increase social motivation and encourage adaptive behaviours aimed at fostering social connections [37], whereas chronic loneliness might diminish social motivation, leading to maladaptive behaviours and social disconnection [66]. How threat learning might be differentially affected by chronic versus acute loneliness remains to be explored. Additionally, the relationship between loneliness and isolation is probably complex and might vary between individuals with different personality types or mental health conditions and across different contexts (e.g. social versus non-social settings; [67–69]).

In our study, although virtual social interactions alleviated self-reported state loneliness, they did not result in significant differences in threat learning between the two isolation sessions (iso-with-media and iso-total). This suggests that virtual social interactions did not remediate the effects of isolation on threat learning. However, the nature of these virtual interactions is probably an important factor. For instance, passive scrolling on social media might have different effects compared with active conversations via video call [70,71], and the identity and relationship between those in the interaction might also play a role. In the current study, we asked only a few general self-report questions about virtual social interactions during the iso-with-media session. Though our sample size was too small to explore this in a meaningful way, we have included descriptive statistics for these questions in the electronic supplementary material, appendix D.

We took substantial measures to minimize boredom in the isolation sessions; however, boredom was indeed still higher in both isolation sessions than in baseline, and higher in the iso-total than in the iso-with-media session. Our exploratory analyses revealed that state loneliness and boredom played contrasting roles in mediating the relationship between isolation and threat learning. To the extent participants felt lonelier in the iso-total compared with iso-with-media session, they showed increased threat learning (controlling for all other state variables). To the extent participants felt more bored in the iso-total compared with iso-with-media session, they showed decreased threat learning (controlling for all other state variables). Thus, state loneliness and boredom had opposing effects. This might explain the lack of differences in threat learning between the two isolation sessions: heightened boredom in the iso-total compared with iso-with-media session might have counteracted the effects of heightened state loneliness (perhaps by decreasing overall levels of arousal or by reducing attention and engagement with the threat learning task; see [72] for a review discussing the functional purpose of boredom). Considering that our participants were confined to predetermined activities in a controlled experimental setting, in real-world situations where state loneliness and boredom are not as highly correlated, for example, where people are allowed to choose their own activities, the impact of isolation on threat learning might be stronger than observed here. However, we believe that loneliness and boredom could be interconnected constructs, which are difficult to disentangle entirely. For many people, it appears to be fundamentally boring to be on one’s own. We speculate that boredom might be a feature of loneliness, though to our knowledge, no direct research has tested this relationship.

Relatedly, researchers have identified two distinct dimensions of arousal: anxious arousal, ranging from calmness to anxiety, and energetic arousal, ranging from tiredness to energy [73]. A recent study in adults found that social isolation led to decreased energetic arousal and increased fatigue in both laboratory and field settings [74]. Future studies should distinctly measure the effects of experimental isolation on both anxious and energetic arousal and investigate how these dimensions relate to threat learning. Further, more research is needed to explore the interrelationships between loneliness, boredom and threat learning.

### 4.1. Limitations

We acknowledge several limitations in our study. Our participants were socially connected adolescents aged 16–19 without diagnosed mental health conditions. Due to our screening criteria, participants could not exhibit high levels of chronic loneliness or have a significantly smaller social network compared with a typical adolescent sample. This approach was implemented in order to avoid exposing vulnerable young people to potentially distressing isolation and to prevent conflating the effects of acute and chronic isolation. Consequently, our sample was probably biased towards non-lonely adolescents with higher social motivation, limiting the generalizability of our findings to other populations. Moreover, there might be substantial individual differences in the amount of social connection needed to prevent loneliness, and this individual variability might have implications for long-term well-being. Future research should prioritize the inclusion of diverse populations, such as those experiencing chronic loneliness, individuals with varying personality types, those with mental health conditions and age groups with lower social motivation compared with typically highly socially motivated adolescents.

While our explicit measures (subjective responses) of threat learning showed clear effects, our implicit measures (electrodermal activity) were weaker (although still significant). While subjective responses are standard in threat learning paradigms and have shown good validity (studies have found differences in threat learning as measured by subjective responses between participants with anxiety versus healthy comparison participants) [46,75,76], there is a possibility that participants’ ratings might have been affected by social desirability or demand characteristics.

Data collection commenced in April 2021 during the COVID-19 pandemic, a period marked by fluctuating social restrictions that might have influenced participants’ baseline levels of social isolation. Despite ongoing social distancing measures, the majority of participants in our study were experiencing some degree of in-person social interaction at the time of testing (schools in England had reopened by this time). Nonetheless, the effect of our experimental isolation manipulation might be amplified in a post-pandemic context in which higher levels of in-person social contact occur.

Finally, our experiment began with a baseline assessment of each participant. This design allowed us to examine the impacts of isolation relative to an unaffected baseline. Therefore, as the baseline was always completed first, there is the possibility our results are due to order effects. The two isolation sessions (iso-with-media and iso-total) were counterbalanced, however, which allowed us to conduct sensitivity analyses to examine whether order effects between the two sessions played a role in our findings. Though these sensitivity analyses did not suggest that order played a role in our results, future research could benefit from counterbalancing the sequence of all sessions. Relatedly, participants underwent MRI scanning before completing the behavioural testing, which could have influenced their behaviour. Future studies should consider including a natural social interaction baseline.

## 5. Conclusion

Social isolation, regardless of access to virtual interactions, resulted in increased self-reported loneliness and heightened threat responses during threat learning in adolescents aged 16–19 years. While self-reported state anxiety did not increase following isolation in our sample, increased threat learning has potential implications for the development of anxiety and other threat-related disorders such as specific phobias, obsessive-compulsive disorder (OCD) and post-traumatic stress disorder (PTSD). It is important to note that our study focused on healthy adolescents without pre-existing mental health conditions. It is possible that the observed effects of isolation on threat learning would be more pronounced in individuals already prone to anxiety or those experiencing chronic loneliness.

Adolescence is a critical period for the onset of many anxiety disorders [77], and anxiety disorders have risen in young people in recent years in many countries [78–80]. It has been suggested that adolescent-specific differences in associative threat learning contribute to the onset of anxiety during this developmental stage, including challenges in extinguishing learned threat responses and retaining that extinction over time [81]. Concurrently, there has been a marked increase in feelings of loneliness among adolescents. Globally, the number of adolescents experiencing heightened loneliness in 2018 was nearly double that of 2012, with a more pronounced increase among girls compared with boys [7]. These vulnerabilities make adolescence a particularly sensitive period for the consequences of heightened threat learning, which might be exacerbated by isolation and loneliness. Understanding these dynamics is crucial for developing effective interventions to mitigate the long-term impact of social disconnection on mental health.

## Data Availability

Supplementary material (including data, materials, and code) for this study are publicly available at [82]. This study was pre-registered on the Open Science Framework [83].
